# Mature and Precursor Brain-Derived Neurotrophic Factor Have Individual Roles in the Mouse Olfactory Bulb

**DOI:** 10.1371/journal.pone.0031978

**Published:** 2012-02-21

**Authors:** Thomas Gerald Mast, Debra Ann Fadool

**Affiliations:** 1 Department of Biological Science, The Florida State University, Tallahassee, Florida, United States of America; 2 Program in Neuroscience, The Florida State University, Tallahassee, Florida, United States of America; 3 Institute of Molecular Biophysics, The Florida State University, Tallahassee, Florida, United States of America; Federal University of Rio de Janeiro, Brazil

## Abstract

**Background:**

Sensory deprivation induces dramatic morphological and neurochemical changes in the olfactory bulb (OB) that are largely restricted to glomerular and granule layer interneurons. Mitral cells, pyramidal-like neurons, are resistant to sensory-deprivation-induced changes and are associated with the precursor to brain-derived neurotrophic factor (proBDNF); here, we investigate its unknown function in the adult mouse OB.

**Principal Findings:**

As determined using brain-slice electrophysiology in a whole-cell configuration, brain-derived neurotrophic factor (BDNF), but not proBDNF, increased mitral cell excitability. BDNF increased mitral cell action potential firing frequency and decreased interspike interval in response to current injection. In a separate set of experiments, intranasal delivery of neurotrophic factors to awake, adult mice was performed to induce sustained interneuron neurochemical changes. ProBDNF, but not BDNF, increased activated-caspase 3 and reduced tyrosine hydroxylase immunoreactivity in OB glomerular interneurons. In a parallel set of experiments, short-term sensory deprivation produced by unilateral naris occlusion generated an identical phenotype.

**Conclusions:**

Our results indicate that only mature BDNF increases mitral cell excitability whereas proBDNF remains ineffective. Our demonstration that proBDNF activates an apoptotic marker *in vivo* is the first for any proneurotrophin and establishes a role for proBDNF in a model of neuronal plasticity.

## Introduction

The loss of sensory afferents (i.e. inputs) induces cell death in target brain regions or nuclei [1]–[5]. The neurons that die following sensory deprivation differ across modalities and are tissue-specific. Visual [2] and auditory [3] nuclei respond to sensory loss by losing relay or projection neurons. Interneurons die following loss of sensory input to the spinal cord dorsal horn [4] and to the olfactory bulb (OB) [1], [5]–[7]. OB interneuron morphologic degeneration can be induced by both deafferentation (direct insult to the peripheral neuron) [6], [8] and by sensory deprivation (indirect insult by loss of odorant stimulation) [5], [7], [9], [10]. Olfactory sensory deprivation via unilateral naris occlusion was developed to better elucidate the regulation of neonatal OB development [9], [11], and has also been a useful model of neuronal insult in the adult mouse [6], [7], [10], [12], [13].

Mouse OB interneurons are differentially sensitive to sensory deprivation induced by unilateral naris occlusion. Catecholamine interneurons, in the glomerular layer, have been shown to be particularly sensitive to naris occlusion. In the odor-deprived bulb, relative to the contralateral control OB, catecholamine loss has been measured by lower dopamine concentration [9], [12], [14], lower TH expression and activity [12], loss of tyrosine hydroxylase (TH) immunoreactivity [6], [10], and also by increased DNA fragmentation in the glomerular layer [5]. Thus there is evidence for a loss of cellular phenotype and for total cell death. The mechanism underlying this interneuron loss in the mouse OB is still unknown. Following sensory deprivation granule cells, the other major class of OB interneurons, express activated-caspase 3 [7], an enzyme involved in neural plasticity and apoptosis. OB catecholamine neurons have not been shown to express activated-caspase 3 following loss of sensory input, however, these neurons are immunoreactive for activated-caspase 3 prior to methamphetamine-induced apoptosis [15].

Previously we investigated the role of brain-derived neurotrophic factor (BDNF) following OB sensory deprivation. BDNF immunohistochemistry revealed a selective loss of BDNF-immunoreactive fibers from the most superficial extent of the external plexiform layer [13]. This layer is known to shrink in size following naris occlusion [11], [16] and contains processes of glomerular interneurons [17]. At the same time, mitral cells appeared to gain BDNF immunoreactivity. We also found that the major BDNF isoform in the OB was the unprocessed BDNF precursor, proBDNF [13]. Neurotrophins are secreted as ‘pro’ isoforms (i.e. proBDNF, proNGF) [18] and require processing by an extracellular protease that will cleave the pro domain from the mature neurotrophin. ProBDNF has been considered an inactive precursor prior to cleavage by the serine protease plasmin and other selective matrix metalloproteinases [18]–[21]. The activated or mature BDNF can then serve as a ligand for the tropomyosin-related kinase B (TrkB) receptor and promote cell survival and synaptic plasticity [19]. However, proBDNF can also directly bind to and activate TrkB [22]. All proneurotrophins have higher affinities for the p75 neurotrophin receptor (p75NTR), which lacks a kinase domain, than for their respective Trk receptors [18], [23], [24]. Proneurotrophin activation of p75NTR can induce cell death [18], [23]–[26]. It is unknown if proneurotrophins can induce cell death *in vivo*. Interestingly, mouse mitral cells express TrkB [27], [28], but do not express p75NTR [16], [27] and respond to BDNF stimulation with a reduction in voltage-gated potassium current *in vitro* [29], [30]. The molecular targets for BDNF-induced current suppression are three tyrosine residues on the N- and C- terminal aspects of the Kv1.3 channel that become phosphorylated [29]. OB glomerular layer interneurons express p75NTR [16], [27] but not TrkB [27]. While suppression of Kv1.3 would be predicted to increase mitral cell excitability, current-clamp studies have never been performed. Our goal, therefore, was to investigate a direct action of mature versus proBDNF on mitral cells via activation of the TrkB receptor kinase.

In the present study we hypothesized that proBDNF alters adult mouse OB function both acutely and chronically by action on two different neurotrophin receptors expressed in different neuronal types. Acute effects by proBDNF were predicted to be mediated by the mitral cell TrkB receptor and could be measured by changes in electrical excitability. Conversely, proBDNF chronic effects were predicted to be mediated by p75NTR as expressed in the glomerular interneuron and could be measured by an increase in activated-caspase 3 immunoreactivity and a loss of TH expression.

## Results

### BDNF, but not proBDNF, increases mitral cell excitability

**Figure 1:**
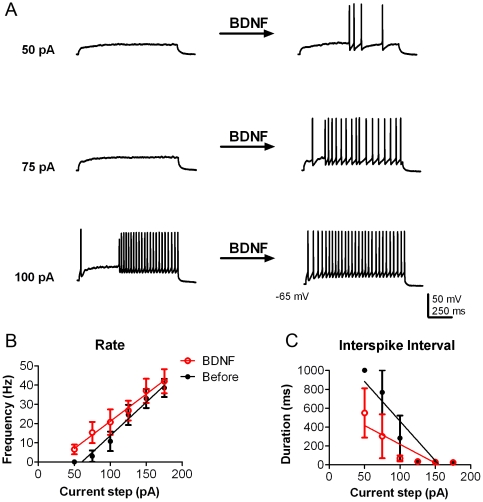
BDNF increases mitral cell excitability. (A) Representative action potential trains in a mitral cell in response to various current injections of 1000 ms duration before (left) and approximately 10 min after 10 ng/ml BDNF bath application (right). The resting membrane potential was held near −65 mV. (B) Line graph of the mean (± s.e.m.) spike frequency (Hz) evoked at each current injection as recorded in A. Closed circles (black) represent data before BDNF stimulation and open circles (red) represent the same neurons after BDNF stimulation. (C) Line graph of the mean (± s.e.m.) interspike interval (ISI) at each current injection. Data were fit with linear regressions to facilitate visualization. BDNF had a significant treatment effect for both rate and ISI; two-way ANOVA (N = 4; p<0.05).

Voltage-clamp studies have established that BDNF-evoked activation of TrkB causes current suppression of mitral cell outward current attributed to multiple phosphorylation of the predominant voltage-gated potassium channel, Kv1.3 [28]–[32]. Since the mouse OB expresses more proBDNF than it does mature BDNF [13], we sought to determine if both proBDNF and BDNF were active ligands in the mouse OB. Mitral cells were identified in OB slices as large, pyramidal cells in a discreet layer when viewed with infrared optics [33]. BDNF bath application modulated the threshold to the first action potential so that 4 out of 4 mitral cells fired at a lower intensity of current injection; measured as an average of 100 pA before versus that of 69 pA following BDNF addition (Figure 1A). Overall, mitral cells significantly increased spike firing frequency (the total number of spikes per 1s sweep or Hz) after BDNF bath application (N = 4, BDNF treatment effect, two-way ANOVA, α≤0.05) (Figure 1B). Mitral cells undergo the firing of intermittent clusters of action potentials (APs), defined as a spike cluster of three or more APs separated by pauses of less than 100 ms [33], [34], which were observed despite the short current injections of our experimental design (Figure 1A). Due to the intrinsic membrane properties of these neurons and our shorter current injection paradigm, we elected to also measure the time between spikes (interspike interval, ISI) as calculated across the duration of the current injection. The duration of the ISI was significantly shorter after BDNF stimulation (N = 4, BDNF treatment effect, two-way ANOVA, α≤0.05) (Figure 1C).

**Figure 2:**
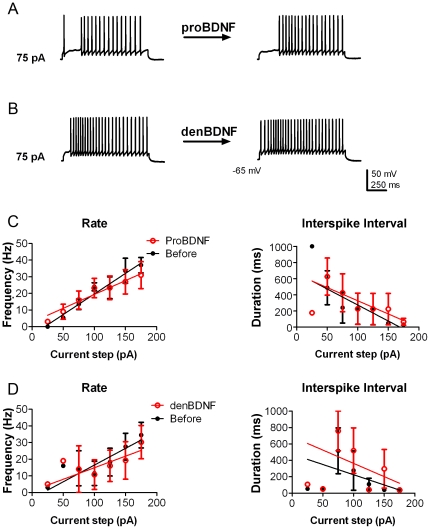
Neither proBDNF nor denatured BDNF increase mitral cell excitability. (A–B) Representative action potential trains in mitral cells in response to 75 pA current injections of 1000 ms duration before (left) and approximately 10 min after (right) bath application of either 10 ng/ml proBDNF (A) or 10 ng/ml denatured BDNF (B, denBDNF). The resting membrane potential was held near −65 mV. (C–D) Line graphs of the mean (± s.e.m.) spike frequency (Hz) and of the mean (± s.e.m.) ISI at each current injection as recorded in A and B before (closed circles; black) and after (open circles; red) proBDNF (C) or denatured BDNF (D). Data were fit with linear regressions to facilitate visualization. Neither treatment had a significant effect upon either rate or ISI; two-way ANOVA (N = 4; p≥0.05).

Since proBDNF has been reported to bind to and activate TrkB [22], we thereby tested the precursor for similar modulation as observed for the mature neurotrophin. Unlike BDNF, however, bath application of cleavage-resistant proBDNF did not induce mitral cells to fire an action potential at lower current intensity (1 out of 5; 75 vs 85 pA) nor did proBDNF modulate frequency (Figure 2A, C) or ISI (Figure 2C) (N = 4, proBDNF treatment effect, two-way ANOVA, α≥0.05). Denatured BDNF (BDNF heated to 95°C for 10 minutes) served as a protein control. Application of denatured BDNF failed to increase mitral cell excitability as measured by frequency and ISI (N = 4, denatured BDNF treatment effect, two-way ANOVA, α≥0.05) (Figures 2B and D). None of the treatments (BDNF, proBDNF, and denatured BDNF) significantly altered the latency to first spike (data not shown) nor were any significant at a given current step (*Bonferroni* α≥0.0083).

### BDNF-evoked increase in mitral cell firing is dependent upon Kv1.3 ion channel

**Figure 3:**
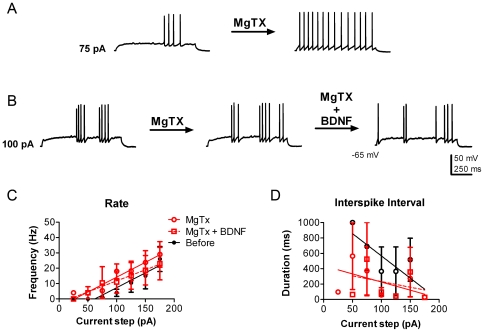
Margatoxin increases mitral cell excitability. (A) Representative action potential trains in a mitral cell in response to a 75 pA current injection of 1000 ms duration before (left) and approximately 10 min after 1 nM Margatoxin (MgTX) bath application (right). (B) Representative action potential train in a mitral cell in response to a 100 pA current injection of 1000 ms duration before (left) and after after MgTX (center) and then after subsequent 10 ng/ml BDNF bath addition (right). The resting membrane potential was held near −65 mV. (C) Line graph of the mean (± s.e.m.) spike frequency evoked at each current injection as recorded in A and B. Closed black circles represent data before BDNF stimulation (Before) and open red circles represent the same neurons after MgTX stimulation (MgTX). Open red squares represent the subsequent application of BDNF while maintaining MgTX block (MgTX+BDNF) as in B. (D) Line graph of the mean (± s.e.m.) ISI at each current injection. Data were fit with linear regressions to facilitate visualization. MgTX has a significant treatment effect for both parameters; two-way ANOVA (N = 5; p≤0.05). BDNF application following MgTX did not significantly alter either rate or ISI; two-way ANOVA (N = 3; p≥0.05).

The Kv1.3 channel carries approximately 40–60% of the outward current in mitral cells, as demonstrated by gene-targeted deletion or pharmacological block [29], [32], [35]. BDNF modulation of Kv1.3 current is prevented by pre-treatment with the selective channel pore-blocking peptide, margatoxin (MgTX), using a primary cell culture preparation in conjunction with voltage-clamp experiments [29]. In order to determine that BDNF-evoked increase in AP firing frequency was attributed to Kv1.3 current suppression, we first characterized MgTX stimulation of the mitral cell using the current-clamp configuration of the OB slice. First, mitral cells were stimulated with the same series of current injections before and after bath application of 1 nM MgTX. Similar to the effect of BDNF, 3 out of 4 mitral cells treated with MgTX bath application fired action potentials at a lower intensity of current injection; measured as an average of 131 pA before versus that of 81 pA following MgTX addition. MgTX bath application significantly modulated mitral cell excitability as measured by frequency (N = 5, MgTX treatment effect, two-way ANOVA, α≤0.05) (Figure 3C) and by ISI (N = 5, MgTX treatment effect, two-way ANOVA, α≤0.05) (Figure 3D). MgTX at this concentration partially blocks homomeric Kv1.3 channels, but not heteromeric Kv1.3 channels in non-neuronal cell types [36], [37]. Importantly, MgTX pre-treatment completely blocked BDNF modulation of spike frequency and ISI (N = 3, BDNF treatment effect, two-way ANOVA, α≥0.05) (Figure 3B–D). This demonstrated that BDNF modulation of spike frequency and ISI is dependent upon Kv1.3 channel conductance. Like the neurotrophin treatments, MgTX did not significantly alter the latency to first spike (data not shown) nor was it significant at a given current step (*Bonferroni* α≥0.0083).

### Intranasal delivery of proBDNF mimics sensory deprivation evoked by naris occlusion

**Figure 4:**
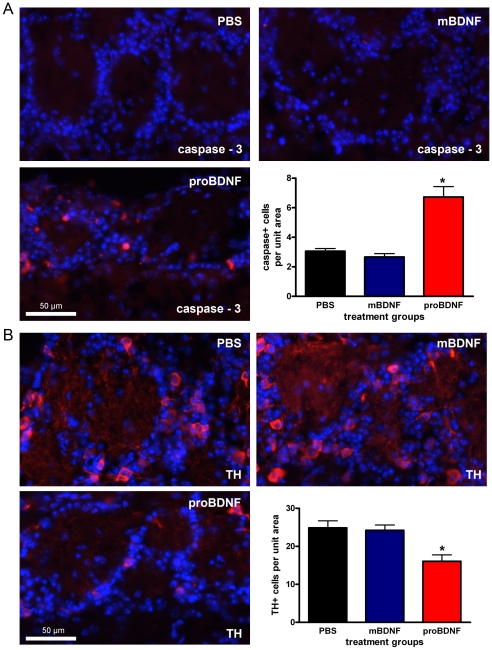
ProBDNF intranasal delivery (IND) increases activated-caspase 3 immunoreactivity and reduces tyrosine hydroxylase immunoreactivity. (A) Representative photomicrographs taken of the mouse glomerular layer following a five day treatment of: PBS, mature BDNF, or proBDNF as administered by IND. Cryosections were incubated with activated-caspase 3 antiserum (1∶100) (red) and with DAPI nuclear stain (blue) to visualize immunoreactive glomerular cells. Bar plot of the mean (± s.e.m.) number of activated-caspase 3 immunoreactive glomerular cells per treatment condition per 93,500 µm^2^ field of view. (B) Same as in A but cryosections were incubated with tyrosine hydroxylase (TH) antiserum (1∶4,000) (red). * = Significantly-different by a one-way ANOVA with a Student-Newman-Keuls post-hoc test (*snk*) (N = 3, α≤0.05).

Since proBDNF failed to alter mitral cell excitability in the acute slice preparation, we surmised that it may act through another neurotrophin receptor. Adult mouse OB glomerular layer interneurons, but not mitral cells, express p75NTR [16], [27] and are prone to sensory deprivation-induced cell death [5]. Furthermore, proBDNF can induce apoptosis in p75NTR-dependent manner *in vitro* [26], [38]. The p75NTR lacks a kinase domain but is associated with apoptosis and cellular pruning [23]. Thus, we tested whether chronic application of proBDNF by intranasal delivery (IND) could induce a phenotype reminiscent of cell loss and plasticity in the mouse OB glomerular layer *in vivo*. Adult mice were given PBS, BDNF, or proBDNF IND once a day for five days and then were sacrificed 24 hours after the last treatment [39]. The mice were then fix perfused and the OBs were processed for immunofluorescence with antiserum against the apoptotic and plasticity marker activated-caspase 3 (see Methods). Few activated-caspase 3 immunoreactive glomerular layer interneurons were present following either PBS or mature BDNF treatment (Figure 4A). In contrast, activated-caspase 3 immunoreactive neurons were approximately 3-fold more abundant in the proBDNF treatment group (Figure 4A). The effect of proBDNF treatment was significant as compared to either PBS or mature BDNF treatment (N = 3, one-way ANOVA, *snk*, α≤0.05). The number of activated-caspase 3 positive neurons is not different between the PBS and mature BDNF treatment groups (Figure 4A) (N = 3, one-way ANOVA, *snk*, α≥0.05). Next, we immunolabeled adjacent sections with an antiserum directed against TH in order to identify glomerular catecholamine interneurons (see Methods). TH immunolabeling could be seen throughout the glomerular layer following treatment with PBS, BDNF, or proBDNF (Figure 4B). However, compared to both PBS and mature BDNF, proBDNF significantly reduced the number of TH immunoreactive glomerular layer interneurons soma (N = 3, one-way ANOVA, *snk*, α≤0.05) (Figure 4B). Thus, proBDNF simultaneously reduces TH immunoreactivity and enhances activated-caspase 3 immunoreactivity.

**Figure 5:**
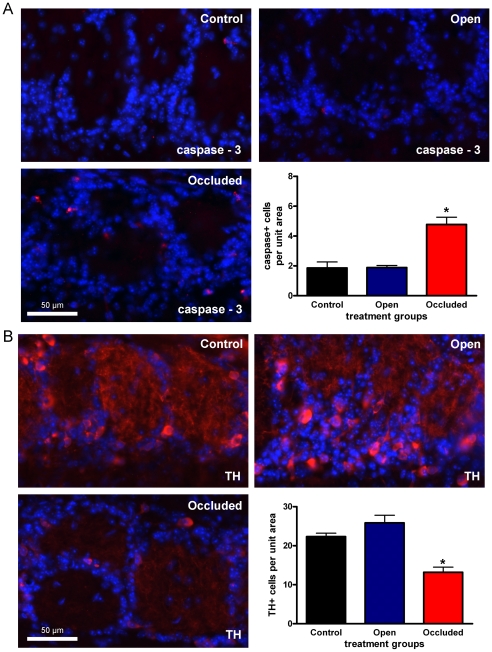
Five days of naris occlusion increases activated-caspase 3 immunoreactivity and reduces tyrosine hydroxylase immunoreactivity. (A) Representative photomicrographs taken of the mouse glomerular layer following five days of sham (control), contralateral to the naris occlusion (open), and ipsilateral to the naris occlusion (occluded) treatment. Cryosections were incubated with activated-caspase 3 antiserum (1∶100) (red) and DAPI nuclear stain to visualize immunoreactive glomerular cells. Bar plot of the mean (± s.e.m.) number of activated-caspase 3 immunoreactive glomerular cells per treatment condition per 93,500 µm^2^ field of view. (B) Same as in A but cryosections were incubated with tyrosine hydroxylase (TH) antiserum (1∶4,000) (red). * = Significantly-different by a one-way ANOVA with a Student-Newman-Keuls post-hoc test (*snk*) (N = 3, α≤0.05).

Next we sought to compare the activated-caspase 3 and TH immunoreactivity seen following IND to a model of neuronal loss and plasticity, olfactory sensory deprivation (Figure 5). Similar to the IND treatment groups, adult mice underwent 5 days of naris occlusion. Prior to sacrifice, animals were visually confirmed to have complete naris closure [11], [13]. As before, we first investigated activated-caspase 3 immunoreactivity. Here the treatment groups consisted of sham (control), contralateral to the nariso cclusion (open), and ipsilateral to the naris occlusion (occluded). Activated-caspase 3 immunoreactive neurons were significantly more abundant in the glomerular layer following naris occlusion as compared to either the control or the open treatment groups (one-way ANOVA, *snk*, N = 3, α≤0.05). TH immunoreactivity was robust following control and open treatments, but weak following naris occlusion (Figure 5B). Naris occlusion significantly reduced TH immunoreactivity in the OB glomerular interneuron soma (N = 3, one-way ANOVA, *snk*, α≤0.05) (Figure 5B). To control for any possible OB shrinkage following either naris occlusion [11] or IND, the perimeters of three OB sections, from which the 12 fields of view were taken, were traced and the total area (mean ± s.e.m.) was calculated as the sum of these sections (see Methods). Naris-occlusion treatment—control (102.8±5.4 mm^2^), open (110.3±7.3) and occluded (101.9±8.6)—had no significant effect on OB area (N = 3, one-way ANOVA, *snk*, α≥0.05). Similarly, none of the IND treatments—PBS (107.1±8.0 mm^2^), mature BDNF (102.3±1.1), and proBDNF (97.4±7.7)—significantly altered OB area (N = 3, one-way ANOVA, *snk*, α≥0.05).

## Discussion

ProBDNF is an active ligand in the mouse olfactory bulb *in vivo*. This novel finding was demonstrated when proBDNF IND produced a phenotype similar to that seen following short-term adult naris-occlusion. Both treatments decreased TH and increased activated-caspase 3 immunolabeling in glomerular layer interneurons. This is the first demonstration of a proneurotrophin inducing a marker of apoptosis *in vivo*. In the OB slice preparation, mitral cells were electrically stimulated via a whole-cell patch-clamp electrode. Bath application of BDNF, but not proBDNF, increased mitral cell spike frequency and initiated spike firing at lower amplitude current injections. Similarly, MgTX mimicked the effect of BDNF on mitral cell spike frequency and initiated spike firing at lower amplitude current injections. Given that MgTX selectively blocks Kv1.3 channels, the mechanism of BDNF modulation of mitral cell excitability is likely via Kv1.3 current suppression that has been shown to be mediated by TrkB-induced phosphorylation of residues Y137 and Y449 in the N- and C- terminal aspects of the Kv1.3 channel [29]. Evoked potassium currents in cultured mouse and rat mitral cells are reduced by ∼35% after BDNF application [29], [30]. Moreover, cultured mitral cells pre-treated with MgTX do not respond to subsequent BDNF stimulation [29], [30], an effect reproduced here in the brain slice preparation. In fact, even though Kv1.3 −/− mice exhibit a 7 fold increase in TrkB expression, application of BDNF to cultured mitral cells remains ineffective to suppress outward current due to lack of the channel target [32].

Here we measured the effect of BDNF isoforms on mitral cells in a slice preparation. It was interesting that a physiological concentration of BDNF (10 ng/ml) [40], [41] increased mitral cell responses to low amplitude current injections but not to larger amplitude current injections. The fact that BDNF had a stronger effect at lower amplitude current injections may be a dose-dependent property in common with other receptor tyrosine kinases. Activation of the insulin receptor kinase allows mitral cells to increase spike frequency and spike adaptation at higher amplitude current injections [33]. The fact that the unprocessed BDNF isoform, proBDNF [18], is more abundant in the mouse OB [13], [16], yet did not alter mitral excitability at any amplitude current injection indicates that mitral cells *in vivo* are likely to be surrounded by suitable concentrations of mature BDNF. The total amount of OB BDNF is still low in comparison to that measured in other brain regions [16], [40], [42]. We conjecture that plasminogen and tissue plasminogen activator—the enzymatic system that cleaves proBDNF in the hippocampus [21]—are expressed in the extracellular space around mitral cells creating local gradients for both proBDNF and mature BDNF.

Our pharmacological results corroborate data from the Strowbridge Laboratory, that demonstrate that low concentrations of 4-aminopyridine (4-AP), a broad potassium channel blocker [43], [44], increases mitral cell firing frequency and burst length [34]. Electrically-stimulated mitral cells generate sub-threshold oscillations that involve the slow-inactivating potassium current [34], [45]. The slow-inactivating potassium current I*_D_* is associated with Kv1.3 [34], [35], [46]. 4-AP is thought to increase mitral cell evoked activity and burst length through channels containing Kv1.3 and other *Shaker* subunits [34]. MgTX was cloned from scorpion venom and was found to maximally inhibit Kv1.3 currents at 110 pM [47]. One nM MgTX has been reported in non-neuronal cells to partially block Kv1.3 homomeric channels and not heteromeric Kv1.3 channels [36], [37] and has been used to block homomeric Kv1.3 currents in cultured neurons [48], [49]. The combinations of *Shaker* alpha subunits expressed by mitral cells, however, are currently unknown. Pharmacological studies have demonstrated that 1.0 nM MgTX can also inhibit Kv1.1 currents [50], but has no effect on Kv1.2 [51], Kv1.4 [51], Kv1.5 [37], [51], Kv1.6 [47], [51], and Kv1.7 and Kv1.8 [51] currents. Furthermore, in tissue preparations, 1.0 nM MgTX has been used to selectively separate Kv1.3 currents in choroid plexus [52], 5 nM MgTX has been used on aspiny forebrain neurons [53], and 10 nM MgTX has used to increase presynaptic excitability in the calyx of Held [54]. It is interesting to speculate that cells with a modest response to 1.0 nM MgTX (i.e. Figure 3B) would be strongly inhibited by a higher concentration that also blocks heteromeric Kv1.3 channels, providing evidence for multiple *Shaker* currents. Other work in our laboratory suggests that Kv1.3 forms heteromers with other *Shaker* subunits in the OB, which are potentially modulated by adaptor proteins and phosphorylation [55]. Therefore, heterogeneity in mitral cells might exist based either upon Kv subunit stoichiometry or channel interactions with adaptor proteins, or a combination of both. It has been recently proposed the heterogeneous responses of mitral cells to current injections may add to neural coding of odors [56]. Varying the composition of *Shaker* channels is a potential mechanism used by mitral cells to achieve heterogeneity.

Unlike potassium channel pharmacological data previously reported in mitral cell slices, we did not see any changes in the latency to first evoked action potential [34]. This is likely due to differences in experimental design. In the previous study, mitral cells were stimulated with longer current injections (5s) and thereby the resulting latencies (800 to 1200 ms) were nearly as long, or longer than, our total pulse duration (1s) and were also highly variable (±150 ms) [34]. Therefore, it is not surprising that no effect on the latency to first evoked action potential was observed for our study in which the pulse duration was likely too short to accurately access this variable.

When proBDNF failed to increase mitral cell excitability we explored an alternative function in the glomerular layer. Sensory deprivation via naris occlusion reduces the number of proBDNF immunoreactive glomerular cells [13] and can induce cell death in the OB glomerular layer [5]. Glomerular interneurons express the p75NTR [16], [27] that can induce apoptosis [23]. Both proBDNF and pro-nerve growth factor (proNGF) have a higher affinity for the p75NTR than for their respective Trk receptors [18], [23], [24]. The increased affinity for p75NTR by proneurotrophins is likely due to binding to the p75NTR co-receptor sortilin [23]–[26]. Regardless of the ligand-receptor interaction, both proBDNF and proNGF have been shown to induce p75NTR dependent cell-death. ProNGF has been shown to induce p75NTR-dependent apoptosis in smooth muscle cells, sympathetic neurons, and in a pheochromocytoma cell line [18] *in vitro*. ProNGF immunoreactivity precedes cell death in oligodendrocytes in an *in vivo* model of nerve damage [25]. Examining the glia in the dying nerve at multiple time points reveals that the oligodendrocytes first upregulate p75NTR and caspase 3 before succumbing to apoptosis [25], indicating a causal relationship. Our data show that application of proBDNF, but not BDNF, induces a proteolytic enzyme involved in apoptosis and plasticity (activated-caspase 3) [7], [15], [23], [25], [39], [57], [58] in the glomerular layer and represents the first demonstration that application of a proneurotrophin activates an apoptotic marker *in vivo*. Interestingly, proBDNF reduced the number of TH immunoreactive cells in the glomerular layer in a manner that mimicked the phenotype of adult naris-occlusion [6], [12], [59]. The same effective proBDNF dose used in our studies (10 ng/ml; 0.2 nM), also induced apoptosis in sympathetic ganglion neurons [26] and cerebellar granule neurons [38] *in vitro*.

Previous studies have also demonstrated a reduction in the number of TH immunoreactive glomerular interneurons in response to adult naris-occlusion [6], [12], [59]; additionally, we report that short-term naris-occlusion enhanced activated-caspase 3 immunoreactivity. Thus, both short-term naris-occlusion and proBDNF administration reduces TH and enhances activated-caspase 3 immunoreactivity. Activated-caspase 3 immunoreactivity following naris occlusion supports previous studies demonstrating neuronal degeneration in the mouse [6] and DNA fragmentation in the rat [5] in the glomerular layer following deafferentation and sensory deprivation, respectively. In contrast, mouse naris-occlusion has been reported to only reduce TH expression and not to induce cell loss [12]. Anecdotally, mouse olfactory nerve axotomy does not induce DNA fragmentation in mouse glomerular layer interneurons [60]. Recently, deafferentation was reported to reduce the number of TH immunoreactive neurons, but not the total number of glomerular interneurons [59]. This conflict may be due to differences in the age of mice. We used young adult mice in the IND experiments in order to keep the age difference between the animals used in the physiology and histology experiments at a minimum. The result was that our animals were about 3 weeks younger than those used by the other groups [12], [59]. Alternatively, activated-caspase 3 may be a mechanism by which sensory input, or lack thereof, regulates synapse and dendrite plasticity [58]. Indeed, after sensory input loss the OB undergoes extensive synaptic remodeling and neurite pruning as measured at the histological, receptor and physiological level [6], [13], [30], [61]–[63]. Furthermore, proliferating neurons of the olfactory bulb, including those in the glomerular layer, briefly express activated-caspase 3 [64]. Therefore, activated-caspase 3 may not be functioning as an apoptotic marker, but instead as a plasticity marker in the mouse glomerular layer.

Our data provide a role for proBDNF in the mouse olfactory bulb and have potentially important implications for therapies that target diseases of neuronal death. Multiple studies have shown that BDNF improves locomotor ability in rodents following experimental ischemia-induced neuronal loss [65]–[67]. ProBDNF levels are reduced in the cortices of human patients that are developing and living with Alzheimer's disease, a condition of massive neuronal loss [68]–[70]. Strikingly, in another human disease of progressive neuronal death, amyotrophic lateral sclerosis (ALS or Lou Gehrig's Disease), intravenous infusions of BDNF were shown to improve the respiratory capabilities of late-stage patients [71]. Intravenous application of BDNF, nonetheless, is inefficient due to the poor blood brain barrier permeability of BDNF and to deleterious side effects [71]. As a result, small molecule ligands for p75NTR and TrkB are being developed as therapeutic agents for diseases of neuronal death [70], [72]. The mouse OB is an exciting model to investigate BDNF and proBDNF actions *in vivo* on easily identifiable, central nervous system neurons that express either p75NTR or TrkB, but not both.

## Materials and Methods

### Ethics statement

All experiments described in this report were approved by the Florida State University Institutional Animal Care and Use Committee (IACUC) under protocol #9912 and were conducted in accordance with the American Veterinary Medicine Association (AVMA) and the National Institutes of Health (NIH).

### Animal Care

All mice (strain C57BL6/J) were housed at the Florida State University vivarium in accordance with institutional requirements for animal care and were maintained on a standard 12/12 hour light/dark cycle. Both male and female mice were incorporated into our experiments and were allowed access to food and water *ad libitum*.

### Antisera and neurotrophins

The rabbit-activated caspase-3 antiserum was used at a final dilution of 1∶100 (Promega, Madison, WI, USA) and the mouse tyrosine hydroxylase antiserum was used at 1∶4,000 (ImmunoStar, Hudson, WI, USA). Neurotrophins were made as a 1 µM stock solution in 0.1% bovine serum albumin (BSA) in phosphate-buffered saline (PBS) and frozen as individual aliquots at −80°C until use. Recombinant cleavage-resistant, mouse probrain-derived neurotrophic factor (proBDNF, B-243) and recombinant human BDNF (B-250) were from Alomone (Alomone, Jerusalem, IL). PBS was made as previously [30]. All other reagents were acquired from Sigma-Aldrich or Fisher Scientific (Suwannee, GA, USA).

### Slice preparation

Postnatal day 15–35 (P15–35) mice were anesthetized to a surgical plane using gaseous isoflurane in a bell jar (Aerrane; Baxter, Deerfield, IL). The animals were rapidly decapitated and the OBs exposed by removing the dorsal and lateral portions of the cranium between the cribriform plate and the lambda suture [73]. After removing the dura, the OBs were quickly removed, glued down to a sectioning block with a cyanoacrylate adhesive (i.e. Super Glue) and submerged in oxygenated, ice-cold, sucrose-modified artificial cerebrospinal fluid (ACSF). Sucrose-modified ACSF contained (in mM): 83 NaCl, 26.2 NaHCO_3_, 1 NaH_2_PO_4_, 3.3 MgCl_2_, 0.5 CaCl_2_, 72 sucrose, and 22 D-glucose; 310–320 mOsm, pH 7.3. Horizontal sections (275–350 µm) were cut in oxygenated, ice-cold, sucrose-modified ACSF using a Series 1000 Vibratome. Sections were then incubated in oxygenated, sucrose-modified ACSF at 33°C for 30 minutes and then maintained at room temperature in normal ACSF until needed [74]. ACSF contained (in mM): 119 NaCl, 26.2 NaHCO_3_, 1 NaH_2_PO_4_, 2.5 KCl, 1.3 MgCl_2_, 2.5 CaCl_2_ and 22 D-glucose; 300–310 mOsm, pH 7.3. Prepared slices were maintained in an interface chamber containing oxygenated ACSF for up to 8 hours to retain health of tissue prior to recording.

### Electrophysiology

Membrane voltage was measured in the whole-cell configuration and controlled by pClamp 9 software coupled with an Axopatch 200B amplifier (Molecular Devices, Sunnyvale, CA, USA). The analog signal was filtered at either 2 or 5 kHz and minimally digitally sampled every 100 µs. OB slices were visualized at 10× and 40× using an Axioskop 2FS Plus microscope (Carl Zeiss, Thornwood, NY, USA) equipped with infrared, differential interference contrast detection capabilities (Dage MT1, CCD100). The patch pipettes were fabricated from borosilicate glass (Hilgenberg #1405002, Malsfeld, Germany) to yield pipette resistance ranges from 5–8 MΩ. The OB pipette solution contained (in mM): 135 potassium gluconate, 10 KCl, 10 HEPES, 10 EGTA, 1 MgCl_2_, 0.3 Tris GTP, and 4 MgATP; 285–295 mOsm, pH 7.3. Mitral cell bodies were confirmed by soma size (15–30 µm) in the mitral cell layer [34], [45] and as previously optimized using *Thy1*-gfp transgenic mice [33]. Cells that failed to have a resting membrane potential of at least −50 mV, a stable input resistance of at least 150 MΩ, and repetitive spiking in response to current injection were discarded from analysis due to biophysical indicators of poor health. Resting membrane potential was recorded, and adjusted to −65 mV for consistency between cells [34], [45]. The presented values were not adjusted for the −14 mV calculated junction potential. Cells were continuously perfused with standard ACSF plus synaptic blockers (5 µM NBQX and 25 µM APV; Ascent Scientific, Princeton, NJ) (Ismatec; 2 ml/min). Cells were injected with a series of 10 current steps ranging from −50 to +175 pA for 1000 ms. After establishing baseline evoked responses, the bath solution was changed to one containing: cleavage resistant BDNF (10 ng/ml), proBDNF (10 ng/ml), or margatoxin (MgTX) (1 nM; EMD Chemicals, San Diego, CA, USA). After approximately ten minutes of neurotrophin or toxin bath application, the cells were again injected with the series of current steps. The evoked action potential firing frequency (Hz) and interspike interval (ISI) analyses were computed using Clampfit 9 software (Molecular Devices). Each parameter was calculated for the duration of the one second current step. Analyses were performed prior and subsequent to bath application of neurotrophin or toxin so that a blocked design statistical metric could be applied. Prizm software (version 4, GraphPad, San Diego, CA, USA) was used to determine statistical significance at the 95% confidence level with a factorial (or block designed) two-way analysis of variance (ANOVA) for neurotrophin or toxin treatment effect followed by *Bonferroni* post tests to separately compare the effect at each current step.

### Intranasal delivery

Neurotrophin intranasal delivery (IND) was performed as described previously [75]. Briefly, P 30–35 mice were hand-restrained, placed in a supine position, and given three, ten microliter drops of10 ng/µl BDNF or proBDNF into both nares simultaneously. A 0.01% BSA/PBS neurotrophin diluent was used as a vehicle control (PBS). Mice were held supine for 5–10 sec after delivery to ensure all fluid was inhaled. A total of ninety microliters, or three sequential deliveries, were administered to each mouse per day for 5 days. The IND protocol was conducted at 2 pm (circadian time). Intranasally delivered insulin-like growth factor reaches the OB with an efficiency of 0.11% [76]. Based on this efficiency, the molecular weight of BDNF, and the mass of the mouse brain [77], the effective dose reaching the OB was calculated to be between 0.2 to 4 nM BDNF, which is close to the reported concentration of BDNF in the OB [40], [41]. On day 6 of the experiment, animals were perfused using paraformaldehyde dissolved in phosphate-buffered saline (4% PFA/PBS). The OBs were dissected and post-fixed in 4% PFA/PBS for 4 hours. OBs were cryoprotected by incubation in 30% sucrose/PBS overnight at 4°C and then stored in optimal cutting temperature medium (Sakura, Torrance, CA, USA) at −80°C until further use. Sixteen micrometer coronal sections were cut on a crytostat (Leica CM1850, Wetzlar, Germany), placed onto pre-treated slides (Superfrost Plus, Fisher), and stored at −20°C until use for immunofluorescence microscopy.

### Unilateral-naris occlusion procedures

Postnatal day (P) 30–35 mice were anesthetized with isoflurane and the left naris was cauterized using a heated metal probe inserted 1–2 mm into the nostril as described previously [11], [13], [30]. Scar formation resulted in permanent unilateral naris-closure. Complete closure of the cauterized nostril was confirmed by visual examination under a dissecting microscope at five days following the procedure. Sham animals were anesthetized and then the heated metal probe was placed on the tip of the snout. Naris or sham occlusions were performed at 2 pm (circadian time), a time at which the mice in our colony briefly awaken for a meal.

### Immunofluorescence Microscopy

Frozen sections were air-dried on the bench for 60 minutes, re-hydrated with PBS, and then incubated for 60 minutes in blocking solution (5% normal goat serum/2.5% BSA/0.3% Triton in PBS). After the blocking step, sections were rinsed with 0.3% Triton/PBS and incubated with antiserum diluted in blocking solution at 4°C in a darkened, humidified chamber. The incubation was performed overnight. The immunofluorescence signal was detected with either goat anti-rabbit Cy3 or goat anti-mouse Cy3 secondary antisera diluted in PBS (1∶400; Jackson Immunoresearch, PA, USA). Following three washes in PBS, cells were counter-stained with a nuclear stain by incubating slides for five-minutes in diamidino-phenyindole (DAPI, Fisher) in PBS (1∶20,000), washed again in PBS and coverslipped with Fluoromount G (SouthernBiotech, Birmingham, AL, USA) to prevent photobleaching. The following controls were conducted to ensure the technical and biological relevance of the immunofluorescent images: omission of primary antisera, an IND of vehicle group, a sham naris-occlusion group, and the within animal control comparing the olfactory bulbs from the open and occluded nares from the naris-occluded animals. Omission of the primary antisera abolished immunofluorescence and representative biological controls are shown. Image brightness and contrast were adjusted with Adobe Photoshop CS (Adobe Systems Inc., San Jose, CA) for maximal clarity.

### Anatomical analysis

Three animals of each treatment condition (sham and naris occluded; intranasal delivery of PBS, proBDNF or matureBDNF) were sampled. Immunoreactive glomerular cells (including both peri- and juxtaglomerular cells) within 12 fields of view (each 93,500 µm^2^) per animal were counted using Neurolucida (MicroBrightField, Colchester, VT, USA). These 12 fields of view were from representative sections evenly distributed and separated by approximately 460 µm from the rostral to caudal aspects of the OB (3 sections total). On each section, a field of view was taken from the dorsal, ventral, medial, and lateral glomerular layers of the OB and the depth of focus adjusted once for each field of view to obtain the largest and most clear nuclei as seen with DAPI labeling. Immunoreactive-TH cells had clearly defined soma and perinuclear labeling. Activated-caspase 3 immunoreactive cells had punctate labeling near the nucleus as defined by DAPI staining. The counts from these 12 fields of view were then averaged for the analysis. Neurolucida was used to trace entire OB sections that contained the quantified fields of view. The total area of these sections was calculated by summing the area from each of three sections per animal and per treatment as measured by Neurolucida. Using Prizm software (version 4, GraphPad, San Diego, CA, USA), statistical significance was calculated and determined at the 95% confidence level via a one-way analysis of variance (ANOVA) with a Student-Newman-Kewls (*snk*) post-hoc test to separately compare the cell counts across treatment groups.
